# Anæsthetics—Three Deaths from Chloroform and Ether

**Published:** 1874-12

**Authors:** Geo. B. Harriman

**Affiliations:** Boston, Mass.


### ANÆSTHETICS.—THREE DEATHS FROM CHLROFORM AND ETHER.

REPORTED BY GEO. B. HARRIMAN, D. D. S., M. D., BOSTON, MASS.

Within the last year three deaths have taken place in Boston and vicinity by the administration of sulphuric ether and chloroform. Two of them have occurred while undergoing slight dental operations, the third while inhaling sulphuric ether to have an ovarian abscess discharged.

The first was at the office of Chas. Eastham, M. D., No. 25 Fremont street, Nov. 10, 1873, that of Miss Mary F. Crie. Dr. Eastham, who is one of the oldest dentists in the city, gave in substance the following testimony before the coroner's jury. Have been practicing dentistry since 1840, and have used anæsthetics since they were first introduced, and never had an accident before the present. Mrs. Crie had been his patient for twelve years or more, and had given her gas, chloroform and ether; he advised her to take gas, but she insisted on the ether; he administered a mixture of ether and chloroform, about equal parts in bulk; Mrs. Crie said to him, "give me enough this time sure," but yet he did not fully etherize her. When he took hold of the tooth, she straightened back groaning and screaming as if in pain, she immediately went into a rigid state of spasms, her lips were not very pallid, but her eyes were fixed and glassy; he splashed water in her face, and applied ammonia to her nose, and tried artificial respiration, and used a battery; worked over her until he came to the conclusion that she was past recovery. Mrs. Sawyer, who was present all the time, corroborated Dr. Eastham's statement.

Dr. E. S. Wood submitted a written report of the result of his examination of the mixture, and found that it contained sixty per cent. of ether and forty per cent. of chloroform, by bulk. He said the proportion of deaths by ether were one in 25,000, by chloroform one in 2500, and by the mixture of the two one in 5000. Dr. H. J. Bigelow said, "the real question is, has chloroform, besides this narcotic power, some very poisonous influence which acts upon the system, and has ether such a power? I answer chloroform has, and ether has not; chloroform kills suddenly, and ether can not." The coroner's verdict in the above case was, that Mrs. Crie's "death was caused by inhalation of chloroform administered in a mixture of chloroform and ether by the said Dr. Eastham."

The second case occurred within eight miles of Boston, in the city of Lynn, on Dec. 14th, 1873. Dr. George H. Bixby, a well known and highly respectable physician, was about to perform a surgical operation on Mrs. J. W. Homan; he administered the sulphuric ether to her to operate upon an ovarian abscess. He testified "as he was about to commence operations he noticed that the patient gasped open her mouth, after gasping once or twice she ceased breathing." The coroner's verdict was that "she died from inhalation of ether."

The third case is that of Mr. Charles Linscott, who came to his death on the 29th day of September, 1874, in the office of C. P. Rice, M, D., No. 3 Tremont Row, while undergoing an operation of having a tooth extracted while under the influence of chloroform. Dr. Rice testified before the inquest in substance as follows: Graduated at Bowdon Medical College, and practiced dentistry since 1858, and have given chloroform five hundred times; put twenty or thirty drops of chloroform on a napkin and placed it to his mouth and nose, five minutes after he felt no effect; sent for more chloroform. As soon as his arm fell he attempted to operate, but the patient resisted; then he administered more chloroform, extracted the broken tooth when the patient began to breath heavily, and found his pulse growing slow and weak, beating not over twenty times to a minute, in a few seconds they had entirely stopped, though he breathed several times with a sort of spasmodic jerk; dashed cold water in his face, and used aqua ammonia, and endeavored to inflate his lungs by raising his arms over his head and breathing in his mouth and pressing the air out of his lungs. Did not consider chloroform dangerous in his own practice.

Drs. G. M. Gay and R. V. Hodges testified of the dangerous character of chloroform, and had known of it killing a healthy young woman who had taken only fifteen drops.

The coroner's verdict was that she died from inhalation of chloroform.

